# Gender-based differences in the relationship between fatty liver disease and atherosclerosis

**DOI:** 10.5830/CVJA-2016-014

**Published:** 2016

**Authors:** Hyun-Jin Kim, Chae-Wan Lim, Jae hyuk Lee, Hyung-Bok Park, Yongsung Suh, Yoon-Hyeong Cho, Tae-Young Choi, Eui-Seok Hwang, Deok-Kyu Cho, Hyun-Jin Kim

**Affiliations:** Division of Cardiology, Department of Internal Medicine, Myongji Hospital, Gyenggi-Do, South Korea; Division of Cardiology, Department of Internal Medicine, Myongji Hospital, Gyenggi-Do, South Korea; Division of Cardiology, Department of Internal Medicine, Myongji Hospital, Gyenggi-Do, South Korea; Division of Cardiology, Department of Internal Medicine, Myongji Hospital, Gyenggi-Do, South Korea; Division of Cardiology, Department of Internal Medicine, Myongji Hospital, Gyenggi-Do, South Korea; Division of Cardiology, Department of Internal Medicine, Myongji Hospital, Gyenggi-Do, South Korea; Division of Cardiology, Department of Internal Medicine, Myongji Hospital, Gyenggi-Do, South Korea; Division of Cardiology, Department of Internal Medicine, Myongji Hospital, Gyenggi-Do, South Korea; Division of Cardiology, Department of Internal Medicine, Myongji Hospital, Gyenggi-Do, South Korea; Department of Translational Medicine, College of Medicine, Seoul National University, Seoul, South Korea

**Keywords:** carotid intima–media thickness, fatty liver, athero-schlerosis

## Abstract

**Background:**

Carotid intima–media thickness (CIMT) is a surrogate of subclinical atherosclerosis. Fatty liver disease is also linked to increased risk of cardiovascular events. The aim of this study was to evaluate the association between fatty liver disease and CIMT according to gender.

**Methods:**

Patients who had undergone carotid and abdominal ultrasound between June 2011 and December 2013 were retrospectively evaluated. The differences between the CIMT values measured in the common carotid artery and the prevalence of carotid plaque in patients with fatty liver disease and those with normal livers were investigated.

**Results:**

Out of a total of 1 121 patients, the men had more fatty liver disease than the women. The mean CIMT of the men was significantly higher than that of the women, and the men had more plaque than the women. The women with fatty liver disease had a significantly higher mean CIMT value and more plaque than the women with normal livers. The differences between the men with fatty liver and those with normal livers in mean CIMT values and in the prevalence of plaque were not significant. In the women, multivariate analysis showed that fatty liver disease was independently associated with subclinical atherosclerosis [adjusted hazards ratio (HR) 1.65, 95% confidence interval (CI) 1.007–2.697, p = 0.047].

**Conclusions:**

The men had more fatty liver disease, carotid plaque and higher CIMT values than the women. Fatty liver disease was a useful predictor of atherosclerosis, especially for the female study patients.

## Background

Carotid intima–media thickness (CIMT) is a surrogate of subclinical atherosclerosis and a predictor of cardiovascular events.^1^-^3^ Because CIMT can easily be estimated by ultrasonography, the assessment of CIMT is an effective means of predicting cardiovascular events in asymptomatic patients.^4^ In addition, for predicting cardiovascular risk, measuring the thickness of the intima–media of the common carotid artery plus evaluating whether or not plaque is present on the ultrasound images have been suggested to be a good alternative to ultrasound evaluation of all carotid artery segments.^5^

Fatty liver disease is widely accepted as the hepatic expression of the metabolic syndrome.^6^ Patients with fatty liver disease have an increased risk of cardiovascular events, and it was found to be an independent risk factor of cardiovascular disease.^7^-^9^ The prevalence of fatty liver disease is different between men and women and between younger and older individuals.^10^ A previous study found that the male gender was a risk factor for fatty liver disease.^11^ In addition, there are also gender and age differences in the presence of carotid atherosclerosis.^4^,^5^

Although fatty liver disease is linked to increased risk of cardiovascular events, patients with fatty liver disease confirmed by abdominal ultrasound are not always evaluated with regard to atherosclerosis. To date, there are no specific guidelines stipulating that patients with ultrasound-confirmed fatty liver disease should undergo evaluation for subclinical atherosclerosis.

The aim of this study was to evaluate the association between fatty liver disease and CIMT in patients stratified by gender.

## Methods

**Fig. 1:**
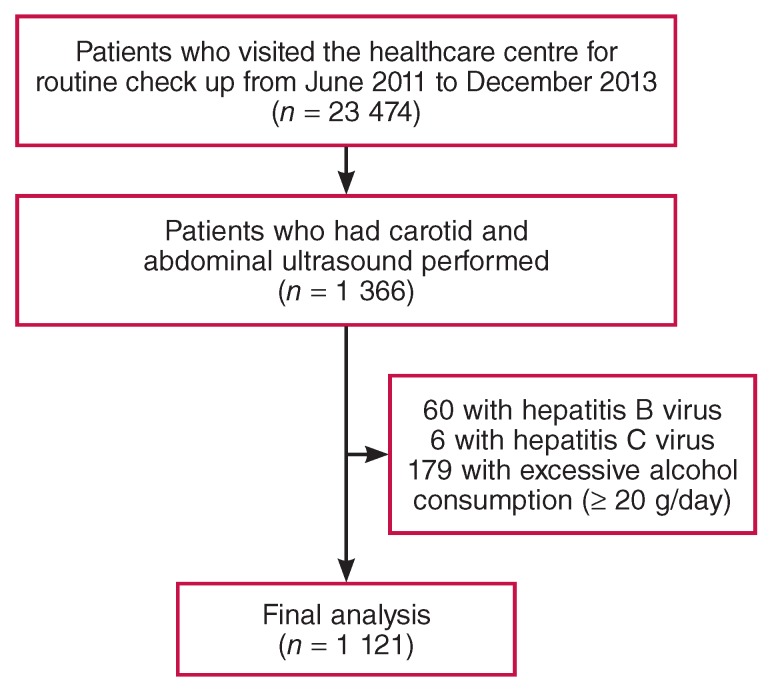
The study population.

Patients who visited the Healthcare Centre of the Myongji Hospital in South Korea for routine check ups between June 2011 and December 2013 were screened retrospectively. Among a total of 23 474 patients considered for inclusion in the study, 1 366 underwent both carotid and abdominal ultrasound. Of these, the following patients with conditions that could lead to chronic liver disease were excluded from the study: 60 patients positive for hepatitis B surface antigen, six positive for hepatitis C antibody, and 179 with excessive alcohol consumption (≥ 20 g/day)^12^ Fig. 1. A total of 1 121 patients were assessed in this study.

The study was approved by the local institutional review board and was conducted according to the Declaration of Helsinki. Written informed patient consent was exempted by the institutional review board.

## Clinical and laboratory assessments

The patients’ demographic and clinical characteristics were reviewed using electronic records. The following were extracted: age, gender, waist and hip circumference, height, weight, history of diabetes, hypertension and dyslipidaemia. Each patient’s body mass index (BMI) was calculated, and obesity was defined as BMI > 30 kg/m^2^.^13^ The waist-to-hip ratio was calculated.

The following laboratory data were extracted: fasting glucose, haemoglobin (Hb) A_1c_, homocysteine, apolipoprotein A-1, apolipoprotein B, total cholesterol, triglycerides, low-density lipoprotein cholesterol (LDL-C), high-density lipoprotein cholesterol (HDL-C), aspartate aminotransferase (AST), alanine aminotransferase (ALT), gamma-glutamyl transpeptidase (GGT) and alkaline phosphatase levels.

## Carotid ultrasound

CIMT measurements of both common carotid arteries were performed using a high-resolution ultrasonography Vivid E9 ultrasound system (GE Healthcare, Little Chalfont, UK) equipped with an 11-l linear transducer. Far-wall mean CIMT measurements of longitudinal images were performed at end diastole in a 10-mm segment located 10 mm proximal to the carotid bulb. Only plaque-free segments of the common carotid arteries were used for CIMT analysis. An experienced ultrasonographer used semi-automated edge-detection software to calculate the mean CIMT value from a single CIMT measurement from the left and a single one from the right common carotid artery, and then averaged the values of the left and right sides.

Carotid plaque was identified as a focal increase in the CIMT of greater than 15 mm or greater than 50% of the surrounding wall.^14^ Both common carotid arteries, the carotid bifurcations, and internal and external carotid arteries were evaluated for plaque. We defined subclinical atherosclerosis as a CIMT value higher than the 75th percentile or the presence of carotid plaque.

## Abdominal ultrasound

Abdominal ultrasound was performed by a different experienced ultrasonographer using an Acuson Sequoia 512 ultrasound system (Siemens Medical Solutions, USA) equipped with a 4-C1 curved transducer. Fatty liver disease (fatty infiltration of the liver) was diagnosed on ultrasound if the liver showed diffuse hyperechogenicity relative to the cortex of the right kidney.^15^ Normal hepatic parenchymal echogenicity was considered to be equivalent to the echogenicity of the cortex of the right kidney.^16^ The study patients were divided into those with fatty liver disease and those with normal livers, based on the ultrasonographic findings.

## Statistical analysis

All categorical data were summarised as frequencies and percentages, and continuous variables are presented as means and standard deviations. The Pearson chi-squared test was used for comparison of categorical variables, and the Fisher exact test was used for comparison of categorical variables with 20% or more of the expected cell frequencies lower than 5. The Student’s t-test was used for comparison of continuous variables, and the Mann–Whitney U-test was used for sample sizes lower than 30 in at least one group.

Linear-by-linear association was also used to determine trends for CIMT and the presence of plaque according to age groups. Univariate followed by multivariate logistic regression analyses were performed to evaluate the association between fatty liver disease and atherosclerosis, with adjustment for individual risk factors, such as age, BMI, hypertension, waist circumference, and triglyceride, HDL-C and fasting glucose levels, which included the components of the metabolic syndrome. A p-value less than 0.05 was considered statistically significant. All data management and analyses were performed using SPSS v 18.0 (SPSS Inc, Chicago, IL).

## Results

A total of 630 men and 491 women (aged 51.7 ± 11.5 and 54.5 ± 11.2 years, respectively) were included in the analysis. Table 1 shows the baseline characteristics of these patients. The men had significantly higher values for waist-to-hip ratio (0.9 ± 0.1 vs 0.8 ± 0.1, p <0.001) and BMI (25.5 ± 3.2 vs 24.5 ± 3.6 kg/m2, p < 0.001) than the women. Systolic and diastolic blood pressures were also significantly higher in the men, and the men had a higher prevalence of diabetes and dyslipidaemia than the women. In addition, the mean fasting glucose levels and liver function test values (AST, ALT, AST/ALT, GGT) were significantly higher in the men than women. There were no significant differences in total cholesterol and LDL-C levels between the men and women, but the triglyceride level was significantly higher in the men.

**Table 1:** Baseline characteristics of the study patients

**	*All (n = 1121)*	*Men (n = 630)*	*Women (n = 491)*	*p-value for difference*
Age (years)	52.9 ± 11.5	51.7 ± 11.5	54.5 ± 11.2	< 0.001
Waist circumference (cm)	81.8 ± 9.4	85.5 ± 7.9	77.2 ± 9.1	< 0.001
Hip circumference (cm)	94.5 ± 6.2	95.4 ± 6.0	93.3 ± 6.3	< 0.001
Waist-to-hip ratio	0.9 ± 0.1	0.9 ± 0.1	0.8 ± 0.1	< 0.001
Height (cm)	163.7 ± 9.1	169.7 ± 6.2	156.1 ± 5.9	< 0.001
Weight (kg)	67.5 ± 12.5	73.6 ± 11.4	59.6 ± 8.9	< 0.001
BMI (kg/m^2^)	25.1 ± 3.4	25.5 ± 3.2	24.5 ± 3.6	< 0.001
SBP (mmHg)	121.8 ± 12.8	122.7 ± 11.7	120.7 ± 14.1	0.011
DBP (mmHg)	74.7 ± 9.8	76.3 ± 9.3	72.7 ±10.0	< 0.001
Previous history				
Hypertension	365 (32.6%)	210 (33.3%)	155 (31.6%)	0.532
Diabetes	155 (13.8%)	104 (16.5%)	51 (10.4%)	0.003
Dyslipidaemia	499 (44.5%)	314 (49.8%)	185 (37.7%)	< 0.001
Fasting glucose (mg/dl)	100.3 ± 21.2	103.7 ± 23.8	96.0 ± 16.3	< 0.001
(mmol/l)	(5.57 ± 1.18)	(5.76 ± 1.32)	(5.33 ± 0.9)	
HbA_C1_(%)	5.8 ± 0.8	5.8 ± 0.9	5.7 ± 0.6	0.058
Homocysteine (μmol/l)	11.2 ± 4.1	12.5 ± 4.5	9.9 ± 3.1	< 0.001
Apolipoprotein A-1 (mg/dl)	142.9 ± 23.9	137.4 ± 22.3	149.2 ± 24.2	< 0.001
Apolipotrotein B (mg/dl)	91.0 ± 21.3	93.3 ± 21.4	88.5 ± 21.0	0.001
Total cholesterol (mg/dl)	191.8 ± 34.4	191.3 ± 34.2	192.4 ± 34.8	0.588
(mmol/l)	(4.97 ± 0.89)	(4.95 ± 0.89)	(4.98 ± 0.9)	
Triglycerides (mg/dl)	138.5 ± 89.3	158.6 ± 99.6	112.8 ± 65.6	< 0.001
(mmol/l)	(1.57 ± 1.01)	(1.79 ± 1.13)	(1.27 ± 0.74)	
LDL-C (mg/dl)	112.7 ± 29.9	113.4 ± 29.8	111.7 ± 30.1	0.356
(mmol/l)	(2.92 ± 0.77)	(2.94 ± 0.77)	(2.89 ± 0.78)	
HDL-C (mg/dl)	49.3 ± 11.6	43.0 ± 9.7	53.5 ± 12.5	21.6 ± 18.5
(mmol/l)	(1.28 ± 0.3)	(1.11 ± 0.25)	(1.39 ± 0.32)	
AST (IU/l)	25.5 ± 12.2	26.6 ± 11.2	24.2 ± 13.3	0.001
ALT (IU/l)	25.5 ± 18.4	28.6 ± 17.7	21.6 ± 18.5	< 0.001
AST/ALT ratio	1.2 ± 0.4	1.1 ± 0.4	1.3 ± 0.5	< 0.001
GGT (IU/l)	42.8 ± 63.0	57.1 ± 78.5	24.3 ± 23.7	< 0.001
ALP (IU/l)	130.4 ± 86.0	132.9 ± 87.6	127.3 ± 83.8	0.283

BMI: body mass index; SBP: systolic blood pressure; DBP: diastolic blood pressure; HbA_1c_: haemoglobin A1c; LDL-C: low-density lipoprotein cholesterol; HDL-C: highdensity lipoprotein cholesterol; AST: aspartate aminotransferase; ALT: alanine aminotransferase; GGT: gamma-glutamyl transpeptidase; ALP: alkaline phosphatase.

**Fig. 2:**
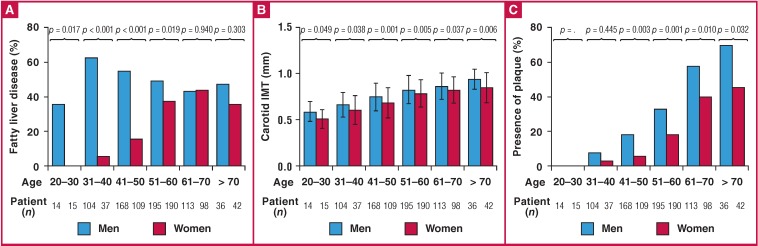
Mean carotid intima–media thickness (CIMT), presence of carotid plaque and fatty liver disease, stratified by gender and age. (A) The prevalence of fatty liver disease was significantly different between men and women under the age of 60 years. (B, C) The mean CIMT values and prevalence of carotid plaque tended to increase with age in both men and women. Among all age groups, the men had significantly higher CIMT values and a higher prevalence of plaque than the women.

A total of 472 of 1 121 (42.1%) patients had fatty liver disease. A significantly higher proportion of men than women had fatty liver disease (51.4 vs 30.1%, p < 0.001) (Table 2). Fig. 2A shows the prevalence of fatty liver disease in men and women, stratified by age. A significantly higher proportion of men than women aged 60 years and under had fatty liver disease. There was no difference in the prevalence of fatty liver disease between the male and female patients aged older than 60 years.

**Table 2:** Gender differences for carotid atherosclerosis and prevalence of ultrasonographic fatty liver disease

**	*All (n = 1121)*	*Men (n = 630)*	*Women (n = 491)*	*p-value for difference*
CIMT (mm)	0.78 ± 0.17	0.79 ± 0.17	0.76 ± 0.17	0.001
Presence of plaque (n, %)	291 (26.0)	192 (30.5)	99 (20.2)	<0.001
75th percentile CIMT (mm)	0.90	0.92	0.88	–
CIMT ≥ 75th percentile or presence of plaque (n, %)	448 (40.0)	269 (42.8)	179 (36.5)	0.032
Ultrasonographic fatty liver disease (n, %)	472 (42.1)	324 (51.4)	148 (30.1)	<0.001

CIMT: carotid intima–media thickness.

The mean CIMT measurement of men was significantly higher than that of the women (0.79 ± 0.17 vs 0.76 ± 0.17 mm, respectively; p = 0.001) and a significantly higher proportion of men had plaque (30.5 vs 20.2%, p < 0.001). The 75th percentiles for the mean CIMT value of the men and women were 0.92 and 0.88 mm, respectively. In addition, a significantly higher proportion of men than women had a CIMT value higher than the 75th percentile or had plaque (42.8 vs 36.5%, p = 0.032) (Table 2). Fig. 2b and Fig. 2c show the mean CIMT values and prevalence of plaque stratified by gender and age. In all age groups, a higher proportion of men than women had plaque, and men had significantly higher CIMT values. Moreover, the mean CIMT values and rates of plaque deposition showed an increasing trend with age in both men and women (p for trend < 0.001 in both genders for both CIMT and plaque).

The mean CIMT values and the prevalence of carotid plaque were significantly higher in both men and women with a history of hypertension and diabetes, compared to those without hypertension and diabetes (Table 3). In addition, the mean CIMT values and plaque rates were significantly higher in women with a higher waist circumference (≥ 80 cm) than women with lower waist circumferences (< 80 cm), in women with dyslipidaemia than women without dyslipidaemia, and in women with fatty liver disease than women without fatty livers. Similar differences were not seen for the men with and without these conditions.

**Table 3:** CIMT and percentage of subjects with carotid plaques according to binary risk factors

	Men (n = 630)				Women (n = 491)			
*Variable*	*CIMT (mm)*	*p-value*	*Presence of plaque (%)*	*p-value*	*CIMT (mm)*	*p-value*	*Presence of plaque (%)*	*p-value*
Waist circumference		0.101		0.185		< 0.001		0.001
≥ 90 cm (M), ≥ 80 cm (W)	0.81 ± 0.16		26.7		0.80 ± 0.17		27.7	
< 90 cm (M), < 80 cm (W)	0.79 ± 0.17		32.2		0.73 ± 0.17		15.4	
History of hypertension		0.007		< 0.001		< 0.001		< 0.001
Yes	0.82 ± 0.16		42.9		0.81 ± 0.15		35.5	
No	0.78 ± 0.17		24.3		0.73 ± 0.17		13.1	
History of diabetes		0.001		< 0.001		< 0.001		< 0.001
Yes	0.85 ± 0.15		46.2		0.83 ± 0.16		43.1	
No	0.78 ± 0.17		27.4		0.75 ± 0.17		17.5	
History of dyslipidaemia		0.851		0.821		0.016		0.024
Yes	0.79 ± 0.16		30.9		0.78 ± 0.16		25.4	
No	0.79 ± 0.17		30.1		0.74 ± 0.18		17.0	
Fatty liver disease		0.513		0.635		< 0.001		0.001
Yes	0.79 ± 0.17		29.6		0.81 ± 0.17		29.7	
No	0.80 ± 0.16		31.4		0.73 ± 0.17		16.0	
BMI (kg/m^2^)		0.549		0.017		0.465		0.771
> 30	0.78 ± 0.18		16.4		0.78 ± 0.14		22.0	
≤ 30	0.79 ± 0.16		31.8		0.76 ± 0.17		20.0	

CIMT: carotid intima–media thickness; BMI: body mass index.

Table 4 shows the results of univariate and multivariate analysis of factors in relation to subclinical atherosclerosis, stratified by gender. Univariate analysis found that older age, history of hypertension, and high fasting glucose levels were significantly associated with subclinical atherosclerosis in men. Multivariate analysis found that older age [hazard ratio (HR) 1.11, 95% confidence interval (CI): 1.084–1.130, p < 0.001] and high fasting glucose levels (HR 1.01, 95% CI: 1.001–1.018, p =0.034) were independent predictors of subclinical atherosclerosis in men. Univariate analysis showed that fatty liver disease, larger waist circumference, older age and history of hypertension were associated with subclinical atherosclerosis in women. Multivariate analysis found that fatty liver disease was an independent predictor of subclinical atherosclerosis in women (HR 1.65, 95% CI: 1.007–2.697, p = 0.047). Older age (HR 1.08, 95% CI: 1.056–1.107, p > 0.001) and hypertension (HR 1.82, 95% CI: 1.135–2.902, p = 0.013) were also independent predictors of subclinical atherosclerosis in women.

**Table 4:** Univariate and multivariate analysis for risk of subclinical atherosclerosis

	*Men (n = 630)*				*Women (n = 491)*			
	*Univariate analysis*		*Multivariate analysis*		*Univariate analysis*		*Multivariate analysis*	
*Variables*	HR	CI (95%)	HR	CI (95%)	HR	CI (95%)	HR	CI (95%)
Age	1.10	1.082–1.123	1.11	1.084–1.130	1.09	1.068–1.115	1.08	1.056–1.107
BMI (kg/m^2^)	0.99	0.945–1.043			1.04	0.989–1.096		
Waist circumference (cm)	1.01	0.993–1.034			1.04	1.016–1.060		
Hypertension	1.88	1.342–2.628			2.88	1.942–4.276	1.82	1.135–2.902
Fatty liver disease	1.05	0.765–1.440			2.09	1.408–3.102	1.65	1.007–2.697
Triglycerides (mg/dl)	1.00	0.997–1.001			1.00	1.000–1.005		
HDL-C (mg/dl)	1.00	0.982–1.015			0.99	0.972–1.002		
Fasting glucose (mg/dl)	1.02	1.008–1.024	1.01	1.001–1.018	1.01	1.000–1.023		

BMI: body mass index; HDL-C: high-density lipoprotein cholesterol; HR: hazard ratio; CI: confidence interval.

Subclinical atherosclerosis is defined as an increased CIMT value (≥ 75th percentile CIMT) or the presence of plaque.

Although a significantly higher proportion of men aged 60 years and under had fatty liver disease than the women (Fig. 2A), fatty liver disease was significantly associated with increased CIMT or plaque formation in women aged 60 and under (HR 2.13, 95% CI: 1.268–3.562, p = 0.004). The association between fatty liver disease and subclinical atherosclerosis in men aged 60 and under was not significant (HR 1.10, 95% CI: 0.753–1.608, p = 0.620). Moreover, although there was no difference in the prevalence of fatty liver disease between the male and female patients older than 60 years, women with fatty liver disease tended to have subclinical atherosclerosis (HR 1.85, 95% CI: 0.929–3.683, p = 0.080). Fatty liver disease in older men was not associated with subclinical atherosclerosis (HR 1.35, 95% CI: 0.647–2.803, p = 0.456).

## Discussion

The prevalence of fatty liver disease was higher in the men than the women in our study, especially in patients aged 60 years and under. The mean CIMT value was higher and the presence of plaque was more in men than women, regardless of age. Interestingly, a significantly higher mean CIMT value was found in the women with fatty liver disease than in the women with normal livers, and women with fatty liver had more carotid plaque than women with normal livers. Fatty liver disease was independently associated with subclinical atherosclerosis in the women only, which was defined as a higher CIMT value (≥ 75th percentile ≥ 0.88 mm) or presence of carotid plaque.

CIMT values and the prevalence of carotid plaque increased with age for both genders in our study cohort. The differences in CIMT values between genders persisted for all age groups, and the differences in the prevalence of plaque between genders persisted for groups of study patients. These results are consistent with the findings of the Gutenberg Heart study, in which earlyatherosclerosis was more frequent in men than women and was significantly associated with age.^17^ An Asian study performed in Taiwan also found that CIMT was significantly greater in men than women.^18^

A Korean multicentre epidemiological study found carotid plaques in 17% of a healthy population with a mean age of 49 years and in 35% of hyperlipidaemic, hypertensive patients with a mean age of 51 years.^19^ Our study found a similar prevalence of carotid plaques (26.0% of the total study population and 30.5% of men and 20.2% of women). In addition, our results revealed the characteristics of arterial aging and growth of intimal smooth muscle cells that increased with age, along with the presence of vascular plaque.

Along with an earlier study,^20^ our study found that the prevalence of fatty liver disease was significantly higher in men than women. Many factors may contribute to gender differences in the prevalence of fatty liver disease. First, the men had a significantly larger waist-to-hip ratio than the women. The prevalence of fatty liver disease is significantly higher in people with a large waist-to-hip ratio. An increased waist-to-hip ratio is directly correlated with increased visceral adipose tissue, which is associated with hepatic insulin resistance in men; and insulin resistance is also associated with fatty liver disease.^21^,^22^ Other factors, including sex hormones and gender lifestyle differences may also be associated with gender differences in the prevalence of fatty liver disease.^20^ We suspect that the baseline characteristics of men, including factors indicating the presence of the metabolic syndrome, may also have contributed to the higher prevalence of fatty liver disease and carotid plaque, as well as higher CIMT values in the male than in the female participants in our study.

Vascular remodelling, which presents with signs of endothelial dysfunction, increasing thickness of the carotid intima–media, and vascular plaque, is associated with aging.^23^ BMI, metabolic risk factors, including increased waist circumference, blood pressure, glycated haemoglobin level, HDL-C and triglyceride levels, and lifestyle risk factors, including smoking and alcohol consumption, are also associated with increased CIMT and risk of atherosclerosis.^24^,^25^

Because the male participants in our study had many factors at baseline that indicated a significantly worse clinical profile than the female participants, we analysed the association of some of the components of the metabolic syndrome with CIMT values or presence of carotid plaque, stratified by gender. Our study found different gender-based risk factors for increased CIMT or prevalence of carotid plaque. Among the metabolic risk factors of the women, waist circumference, dyslipidaemic status, and fatty liver disease had significantly more effect on the presence of CIMT and carotid plaque than those factors in men. To determine the hazard ratio of fatty liver disease for developing subclinical atherosclerosis, we adjusted these components of the metabolic syndrome for multivariate analysis.

As we have shown, the prevalence of fatty liver disease is lower in women of reproductive age compared to men of the same age. There was no difference in the prevalence of fatty liver disease between women after menopause (older than 60 years) and men of the same age. These findings may be associated with the protective effect of oestrogen, which is an important regulator of lipid metabolism and has a protective effect against the progression of liver steatohepatitis.^26^,^27^

Previous studies found that oestrogen receptor-gene knockout, aromatase knockout, and double oestrogen receptorgene knockout mice displayed elevated triglyceride levels; and mice with congenital oestrogen deficiency developed fatty liver disease.^27^-^30^ We believe that women who have fatty liver disease may have an abnormal oestrogen receptor-signalling pathway associated with the regulation of lipid metabolism. In addition, oestrogen has been known to prevent age-related adverse vascular remodelling via the inhibition of smooth muscle cell proliferation and endothelial dysfunction, and by improving vascular tone.^23^,^31^ Hence, we believe that women with fatty liver disease who have a defective oestrogen receptor-signalling pathway may have endothelial dysfunction and subclinical atherosclerosis.

Our findings demonstrate that carotid artery evaluation for patients with fatty liver disease, especially for women, has an important role. We believe there should be gender-based screening for subclinical atherosclerosis and modification of risk factors for cardiovascular events. Assessment of CIMT, as a surrogate of subclinical atherosclerosis, may help to further predict cardiovascular events in female patients.

Our study has several limitations. The main limitation was that it was a retrospective observational study. Also, our crosssectional study could not infer causality. Third, our data were derived from an Asian cohort at a single institution; therefore, the study findings on the association of fatty liver disease with CIMT may not necessarily be transferable to other ethnicities.

## Conclusion

The men had more fatty liver disease, more carotid plaque and higher CIMT values than the women in our study. Fatty liver disease was a useful predictor of atherosclerosis, especially for the female study patients. Women with fatty liver disease should undergo monitoring by carotid ultrasound for early detection of atherosclerosis and timely protection against cardiovascular events.
